# Leaf‐associated fungal and viral communities of wild plant populations differ between cultivated and natural ecosystems

**DOI:** 10.1002/pei3.10043

**Published:** 2021-03-25

**Authors:** Yuxin Ma, Tania Fort, Armelle Marais, Marie Lefebvre, Sébastien Theil, Corinne Vacher, Thierry Candresse

**Affiliations:** ^1^ Univ. Bordeaux INRAE UMR 1332 BFP Villenave d’Ornon cedex France; ^2^ INRAE Univ. Bordeaux BIOGECO Pessac France; ^3^ Present address: INRA UMRF 20, côte de Reyne Aurillac 15000 France

**Keywords:** community ecology, culturomics, metabarcoding, metagenomics, microbial community, mycovirus, phytovirus, plant, virome

## Abstract

Plants are colonized by diverse fungal and viral communities that influence their growth and survival as well as ecosystem functioning. Viruses interact with both plants and the fungi they host. Our understanding of plant–fungi–virus interactions is very limited, especially in wild plants. Combining metagenomic and culturomic approaches, we assessed the richness, diversity, and composition of leaf‐associated fungal and viral communities from pools of herbaceous wild plants representative of four sites corresponding to cultivated or natural ecosystems. We identified 161 fungal families and 18 viral families comprising 249 RNA‐dependent RNA polymerase‐based operational taxonomic units (RdRp OTUs) from leaves. Fungal culturomics captured 12.3% of the fungal diversity recovered with metagenomic approaches and, unexpectedly, retrieved viral OTUs that were almost entirely different from those recovered by leaf metagenomics. Ecosystem management had a significant influence on both leaf mycobiome and virome, with a higher fungal community richness in natural ecosystems and a higher viral family richness in cultivated ecosystems, suggesting that leaf‐associated fungal and viral communities are under the influence of different ecological drivers. Both the leaf‐associated fungal and viral community compositions showed a strong site‐specificity. Further research is needed to confirm these trends and unravel the factors structuring plant–fungi–virus interactions in wild plant populations.

## 1 INTRODUCTION

Plant leaves represent one of the largest microbial habitats on Earth (Morris, 2001). They host hyperdiverse microbial communities including bacteria, archaea, filamentous fungi, and yeasts, in addition to viruses (Koskella, 2013; Lindow & Brandl, 2003; Vacher et al., 2016; Vorholt, 2012). These microbial and viral communities influence plant health (Arnold et al., 2003; Hacquard et al., 2017; Saleem et al., 2017), plant nitrogen nutrition (Doty, 2017; Fürnkranz et al., 2008; Moyes et al., 2016), ecosystem primary productivity (Laforest‐Lapointe et al., 2017), and biogeochemical cycles (Bringel & Couée, 2015; Morris et al., 2014; Osono, 2006). Despite their potential importance for natural and cultivated ecosystems, our knowledge of leaf microbial and viral communities remains very limited compared to that of root and rhizosphere communities. Studies are needed to better understand the functions of leaf microbial communities (Rosado et al., 2018) and predict their dynamics in response to global change (Laforest‐Lapointe & Whitaker, 2019). Future studies of leaf microbial communities should go beyond the study of bacterial communities and better integrate fungal and viral communities (Laforest‐Lapointe & Whitaker, 2019). The aim of the present study was to deepen the knowledge of leaf‐associated fungal and viral communities in natural and cultivated ecosystems.

Leaf‐associated viruses include viruses infecting plants (phytoviruses), viruses infecting fungi (mycoviruses), and viruses infecting bacteria and archaea (phages) that are associated with plants. A large fraction of plant‐associated viruses in wild plants is composed of double‐stranded RNA (dsRNA) viruses. Several families of dsRNA viruses have both members with plant hosts and members with fungal hosts (mycoviruses). It is therefore difficult to determine whether the dsRNA viruses detected from plant samples are phytoviruses or mycoviruses. The relative proportion of phyto‐ and mycoviruses in plant‐associated viral communities remains unknown (Roossinck, 2015b). In addition, recent reports of naturally occurring cross‐kingdom transmission between plant and fungus further exacerbate this knowledge gap (Andika et al., 2017; Nerva et al., 2017).

Although we know that mycoviruses infect plant‐associated fungi, we do not yet know, in most cases, the effect of these interactions on the fitness of the fungal partner. Mycoviruses can have various effects on their fungal host fitness: many mycoviruses do not induce any obvious phenotypic changes, whereas others can have positive or negative impacts on the fitness and pathogenicity of their host(s) (Ghabrial et al., 2015; Nuss, 2008). Similarly, little is known about the influence of phytoviruses on the fitness of wild host plants under natural conditions. As highlighted by high throughput sequencing (HTS)‐based metagenomic studies, phytovirus diversity has been grossly underestimated so far (Roossinck, 2012; Roossinck et al., 2010; Rosario & Breitbart, 2011; Shi et al., 2016). One of the reasons is that phytoviruses have been traditionally considered as pathogens and, therefore, that most studies have focused on viruses causing visible symptoms in economically important crops (Wren et al., 2006) which represent only a minute fraction of all plant species. There is now evidence that only a fraction of phytoviruses causes obvious disease symptoms (Roossinck, 2005). Indeed, some phytoviruses may even be beneficial to their hosts, providing essential functions in some cases and conditionally beneficial functions in others (Roossinck, 2011). For example, several acute plant viruses may be involved in conditional mutualism by enhancing drought tolerance in plants (Xu et al., 2008).

To deepen the understanding of plant virus diversity, ecology and evolution, recent studies have focused on wild plant populations (Bernardo et al., 2018; Susi et al., 2019; Thapa et al., 2015). Wild plant populations are likely reservoirs of both known viruses and novel ones with a potential to emerge as disease agents (Anderson et al., 2004; Cooper & Jones, 2006; Elena et al., 2014; McLeish et al., 2019; Stobbe & Roossinck, 2014). So far, these studies revealed that infection of wild plants by phytoviruses is quite common and often asymptomatic. They also showed that as a consequence of more extensive efforts in crops virology, novel viruses are more likely to be discovered in wild plants (Roossinck, 2012). The study of viruses at community scale across plant species, ecosystem types and along environmental gradients was initiated very recently (Roossinck, 2015a; Thapa et al., 2015), in comparison to fungal and bacterial communities (for pioneer studies on leaf communities see Cordier et al., 2012; Jumpponen & Jones, 2009, 2010; Redford et al., 2010). Therefore, the environmental factors shaping the composition of plant virus communities remain largely unknown. For instance, a recent study has analyzed viral community composition in six wild plant species from 20 sites over 4 years to test the effects of host identity, location, and sampling year (Thapa et al., 2015). The results showed that only host species significantly influenced community composition. Recent results have also suggested that some viral families may be better adapted to cultivated ecosystems, whereas others could be better adapted to natural ones (Bernardo et al., 2018).

The aim of the present study was to deepen the knowledge of viral and fungal communities associated with a wide range of wild plant species, by combining metagenomics with culture‐based approaches. We collected wild plant leaves in both cultivated and natural ecosystems to (1) test whether viral and fungal communities are richer in natural ecosystems than in cultivated ones, (2) investigate whether the composition of fungal and viral communities varies with ecosystem management and (3) evaluate the proportion of phytoviruses to mycoviruses in leaf samples. These questions were tackled using four subsets of microbial communities, namely the leaf‐associated viral community (encompassing all viruses recovered by applying metagenomics approaches to leaf samples); the leaf‐associated fungal community (all fungi recovered by applying metabarcoding approaches to leaf samples); culture‐associated fungal community (all fungi recovered by culturomics) and the culture‐associated viral community (all viruses recovered by applying metagenomics approaches to fungal cultures).

## 2 MATERIALS AND METHODS

### 2.1 Study sites and sampling design

Herbaceous wild plants and weeds were sampled in 2017 in four sites in southwest France (Figure S1). Two sites (Cul‐1 and Cul‐2) were cultivated, horticultural agro‐ecosystems in which vegetable crops were grown. The Cul‐1 site harbored a large range of crops, including lettuce, spinach, pepper, turnip, whereas the Cul‐2 site mostly had carrots. Two sites (Nat‐1 and Nat‐2) were natural, dry grasslands. The Cul‐1 and Nat‐1 sites were in the Bordeaux area and were sampled in spring. The Cul‐2 and Nat‐2 were in the Bergerac area and were sampled at the beginning of the summer (Figure S1).

At each site, 29 to 40 plants species representing the locally most abundant species were sampled with an identical number of individuals for each plant species (Table S1). At each site, approximately 200 wild plant individuals with adequate stem and leaves were collected and separately stored in plastic bags in the field. Plants were sampled at random among visually healthy plants. Plants with necrotic tissues or with insect infestation were not collected. Crop plants were not sampled in the cultivated sites. Wild plants were identified at the species level in the field and stored in a cool ice chest before being brought back to the laboratory at end of day. Collected plant samples were temporarily preserved in cold storage (4℃) and processed the next day in the laboratory.

### 2.2 Leaf‐associated viral community characterization

#### 2.2.1 Leaf processing and multi‐species pool preparation

**FIGURE 1:**
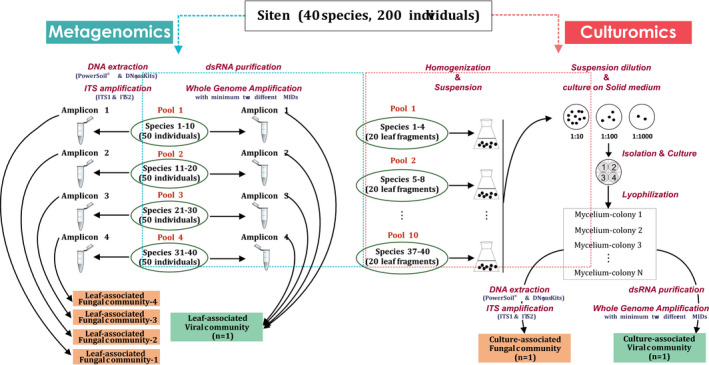
Schematic representation of the sample processing and sequencing strategies for leaf‐associated viral and fungal community analyses based on metagenomics and for fungal culture‐associated viral and fungal community analyses based on culturomics

As the leaf‐associated viral community would be described by site (1 virome per site), for each site, fresh leaf blades of the collected plant individuals were assembled into four multi‐species pools for nucleic acid purification, each pool representing approximately 50 plants (Figure 1; Table S2). All individuals for a given species were allocated to the same pool, so that the four pools of a given site did not share any plant species (Table S2). Each multi‐species pool was assembled using 0.1 g fresh leaf blade of each individual (ca. 5 g in total per pool). The assembled multi‐species pools were subsequently ground into a fine powder in liquid nitrogen for double‐stranded RNA (dsRNA) purification (Figure 1).

#### 2.2.2 Double‐stranded RNAs purification, whole genome amplification (WGA) and Illumina sequencing

Double‐stranded RNAs were purified from each multi‐species pool by two rounds of CF11 cellulose chromatography as described by Marais et al. (2018). A blank control using only reagents was prepared in parallel with every extraction. For conversion to complementary DNAs (cDNA), 3 µl of purified dsRNAs was denatured at 99°C for 5 min and quenched on ice for 2 min. Subsequently, a final volume of 20 µl mixture containing the denatured dsRNAs, 2 mM PcDNA12 (5′ TGTGTTGGGTGTGTTTGGN_12_ 3′), 0.5 mM of dNTPs, 1 U RNase inhibitor, 1× reaction buffer and 200 U Superscript II Reverse Transcriptase (Invitrogen) was incubated at 25℃ for 10 min, at 42℃ for 60 min and at 70℃ for a final 10 min to inactivate the reverse transcriptase. 1.5 U of RNase H was added to the mixture obtained above, which was then incubated at 37℃ for 10 min.

A random whole genome amplification (WGA) was performed using 5 µl of the obtained cDNAs with 1 µM multiplex identifier (MID) primer, 1× reaction buffer, 0.25 mM dNTPs, and 1 U of DyNAzym II DNA polymerase (Finnzymes) in a volume of 50 µl reaction (Marais et al., 2018). The following cycling conditions were used: 94°C for 1 min; 65°C for 0 s; 72°C for 45 s, with a slope of 5°C/s, followed by 30 cycles of 94°C for 0 s; 45°C for 0 s; 72°C for 5 min, with the same slope; and final steps of 5 min at 72°C and 5 min at 37°C (Marais et al., 2018). The above WGA allowed at the same time the tagging of pools from the same site with a specific site‐level MID tag and a minimum of two independent amplifications with different site‐level MID tags were performed for each pool.

PCR products were then purified using the MinElute PCR Purification Kit (Qiagen). Finally, equal amounts (3 µg) of PCR products obtained from each of the four pools corresponding to a sampling site were assembled in one library (resulting in 12 µg PCR products) which broadly integrates the amplified products of 200 sampled plants. The prepared libraries, including negative controls (buffer‐only libraries) were sequenced in multiplexed format (2 × 150 bp) on an Illumina HiSeq 3000 system at the GenoToul sequencing facility (INRAE Toulouse).

### 2.3 Leaf‐associated fungal community characterization

#### 2.3.1 Leaf processing and multi‐species pool preparation

The multi‐species pooling strategy used for leaf‐associated viral community characterization was also applied to describe leaf‐associated fungal community using leaf blades which were dessicated over anhydrous calcium chloride (Table S2).

#### 2.3.2 DNA extractions, ITS1 and ITS2 amplification, and Illumina sequencing

Total DNA was extracted from each multi‐species pool using two different kits: the PowerSoil^®^ DNA Isolation Kit (MO BIO) and the DNeasy Plant Mini Kit (Qiagen), according to the manufacturers’ recommendations (Figure 1).

The libraries were prepared following the protocol provided by Plateforme Génome‐Transcriptome de Bordeaux (PGTB Facility, INRAE ‐ Pierroton, France), which was developed from the Illumina metagenomic sequencing library preparation guide (Illumina, 2013). In detail, the internal transcribed spacer regions (ITS1 and ITS2) were first amplified using tailed primer pairs synthesized with region of interest‐specific primers and overhang adapters in bold underlined: ITS1F (5′‐ **TCGTCGGCAGCGTCAGATGTGTATAAGAGACAG** CTTGGTCATTTAGAGGAAGTAA‐3′) and ITS2 (5′‐ **GTCTCGTGGGCTCGGAGATGTGTATAAGAGACA** GCTGCGTTCTTCATCGATGC‐3′) for ITS1 amplification (Gardes & Bruns, 1993; White et al., 1990); ITS86F (5′‐ **TCGTCGGCAGCGTCAGATGTGTATAAGAGACAG** GTGAATCATCGAATCTTTGAA‐3′) and ITS4 (5′‐ **GTCTCGTGGGCTCGGAGATGTGTATAAGAGACA** TCCTCCGCTTATTGATATGC‐3′) for ITS2 amplification (Op De Beeck et al., 2014). The reaction mixture (25 µl final volume) consisted of 2.5 µl of template DNA, 5 µl of 1 µM of each of the forward and reverse primers, 5 µl of 5X Phusion HF Buffer, 0.5 µl of 10 mM of each dNTP and 2 U of Phusion High‐Fidelity polymerase (Thermo Scientific). PCR reactions were conducted using the following cycling conditions: initial denaturation at 98°C for 30 s followed by 25 cycles at 98°C for 15 s, 59°C for 15 s, 72°C for 30 s with final extension of 72°C for 10 min. The PCR products were purified using platform‐specific SPRI magnetic beads (1X ratio) and quantified using Quant‐it dsDNA Assay kit. MID and Illumina sequencing adapters were added. Libraries were pooled in equimolar amounts using a Hamilton Microlab STAR robot and sequenced on an Illumina MiSeq platform using the MiSeq Reagent Kit v3 (2 × 300 bp). Sequences were cut to 250 bp and demultiplexed with exact index search at the PGTB Facility.

### 2.4 Culturomics: Leaf processing, multi‐species pool preparation, and fungi culturing

To culture out fungi from the collected wild plants, for each individual, a 1 cm^2^ fresh leaf fragment was cut out from the same leaf or from a different leaf from that used for leaf‐associated viral community description. For each site, the leaf fragments were assembled into 10 multi‐species pools (Figure 1), each representing approximately 20 individuals, which were then used for the fungi cultivation using a dilution strategy (Unterseher & Schnittler, 2009). The 10 multi‐species pools of a given site did not share any plant species.

The assembled pools were respectively added to sterile Erlenmeyer flasks containing 15 ml sterile water with 0.1% Tween 20 (Figure 1). The flasks were then incubated at room temperature on an orbital shaker for 20 min before filtering the liquid with sterile gauze. Based on pilot experiments, the filtered solutions were then serially diluted 10, 100 and 1000 times, and 500 μl aliquots were used to inoculate 10 plates of malt agar (MA) and 10 plates of potato dextrose agar (PDA) containing 0.025% chloramphenicol. Plates were incubated at 22℃ and observed regularly for the development of fungal colonies. All developing fungal colonies were isolated from the original plates and transferred to new Petri dishes (4 isolates per plate) containing culture media covered by cellophane in order to facilitate the final collection of mycelia (Figure 1). Grown mycelia (c. 3.5 cm in diameter) were collected, transferred to 50 ml Falcon tubes and lyophilized.

### 2.5 Culture‐associated viral community discovery: dsRNAs purification from fungal cultures WGA and Illumina sequencing

In order to extract dsRNAs for culture‐associated viral community characterization, lyophilized mycelia were assembled in pools (c. 1 mg dry weight per mycelium for a total of c. 0.48–0.64 g dry weight per pool). The protocol for dsRNA purification from dry mycelia, conversion to cDNA and WGA was as described above for leaf‐associated viral communities (Marais et al., 2018). PCR products obtained from each of the pools corresponding to a sampling site were assembled in one library (c. 4 to 12 µg PCR products). The prepared libraries, including negative controls (buffer‐only libraries), were sequenced in multiplexed format (2 × 150 bp) on an Illumina HiSeq 3000 system at the GenoToul platform.

### 2.6 Culture‐associated fungal community discovery: DNA extractions from fungal cultures, ITS1 and ITS2 amplification and Illumina sequencing

The lyophilized mycelia were assembled into several pools, each containing ca. 250 colonies (c. 0.1 mg dry weight per mycelium for a total of 25 mg per pool). For DNA extraction and ITS1 and ITS2 amplification, the same protocols as above were used (Figure 1). PCR products obtained from each of the pools corresponding to a sampling site were assembled in one library, then purified and quantified at the PGTB Facility as described above for leaf‐associated fungal communities. The prepared ITS amplicon libraries from cultures were processed and sequenced in parallel with libraries from leaves.

### 2.7 Bioinformatics for viral community analyses: Reads cleaning, assembly, contigs annotation, and Operational Taxonomic Units (OTU) identification

Viral community analysis was performed using the VirAnnot pipeline v1.0 (https://github.com/marieBvr/virAnnot; Lefebvre et al., 2019). More precisely, the raw sequence reads were first demultiplexed and the MID tags removed using the cutadapt tool v1.9.1 (Martin, 2011). To reduce the cross‐talk between samples caused by index hopping (Illumina, 2017; Van Der Valk et al., 2020), only paired reads with identical MID tags were retained for the next steps. In some cases, to compensate for uneven sequencing depth between libraries, a normalization step was performed by pooling the sequences derived from different replicates and randomly subsampling libraries to the same depth using the seqtk tool v1.2‐r101 (https://github.com/lh3/seqtk). Therefore, one final leaf‐associated viral community and one culture‐associated viral community for each site were generated (Figure 1). The clean paired‐end reads were de novo assembled into contigs using the IDBA‐UD assembler v1.1 (https://academic.oup.com/bioinformatics/article/28/11/1420/266973). Contigs were annotated using BlastN and BlastX (Altschul et al., 1997) against the non‐redundant nucleotide (nt) and protein (nr) GenBank databases (Sayers et al., 2019) with a conservative e‐value cut‐off of 10^−4^. A clustering approach (Lefebvre et al., 2019) was used to define operational taxonomic units (OTUs; Blaxter et al., 2005), following the strategy highlighted by (Simmonds, 2015). Briefly, a search for RNA‐dependent RNA polymerase (RdRp) conserved motifs was performed on all contigs and those encoding a viral RdRp were retrieved and aligned with reference sequences. Distance matrices computed with the ETE3 toolkit v3.1.1 (Huerta‐Cepas et al., 2016) were used to cluster into a single OTU, all contigs diverging by less than 10%. This 10% threshold has been shown to generate, in many viral families, OTUs that are a relatively good approximation of taxonomic species (Lefebvre et al., 2019). This allowed us to generate an OTU table indicating, for each sampling site, the presence/absence and the number of reads corresponding to each OTU.

### 2.8 Bioinformatics for fungal community analyses: Data processing, alpha and beta diversity analyses

The ITS primers were first removed, and the sequences were then filtered, trimmed, merged and chimeras removed using the open‐source software package DADA2 ITS Pipeline Workflow v 1.8 (https://benjjneb.github.io/dada2/ITS_workflow.html) running in R (Callahan et al., 2016) with parameters described in detail in the script (https://doi.org/10.15454/JQPBOW). Taxonomic assignments of Amplicon sequence variant (ASV) were subsequently conducted with the RDP classifier (Wang et al., 2007) embedded in DADA2 and trained with the UNITE general FASTA release for Fungi v8.2 (https://doi.org/10.15156/BIO/786369; Nilsson et al., 2018), with an 80% confidence threshold. The ASV counts, the taxonomy, and sample metadata tables were integrated into one phyloseq object using the Phyloseq package v1.34.0 (McMurdie & Holmes, 2013). Only ASVs assigned to the fungal kingdom were retained for further analyses. Negative controls were used to remove contaminants (as described in Galan et al., 2016). The cross‐contamination threshold (T_CC_) was defined here as the maximal number of sequences of each ASV found in negative control samples. Therefore, four final leaf‐associated fungal communities of four multi‐species pools and one culture‐associated fungal community for each sampling site were generated (Figure 1).

Alpha diversity of fungal communities was described using the community richness (defined as the total number of unique ASVs observed per sample) and the community diversity (measured with the Inverse Simpson's diversity index). These diversity indices were analyzed in R using the Phyloseq package and using the ggplot2 package v3.3.3 for visualization (Wickham, 2016).

We tested the effects of ecosystem management (cultivated/natural), extraction kit (PowerSoil/DNeasy), and sampling region (Bordeaux/Bergerac; Table S3) on community richness and diversity using Generalized Linear Mixed Models (GLMMs). Richness was modeled with a Poisson distribution and diversity was modeled with a Gaussian distribution using the function glmer from the package lme4 v1.1‐26 (Bates et al., 2015). Both models had ecosystem management, extraction kit and their interaction as fixed effects and the sampling region as a random effect. The natural log‐transformed total number of sequences per sample (sequencing depth) was introduced as an offset in all models. Post‐hoc pairwise comparisons were then performed for each level of each factor, with Tukey's adjustment method using lsmeans package v2.30‐0.

Beta diversity representing the fungal community compositional dissimilarities between samples was estimated using binary and quantitative versions of the Jaccard index (Jaccard, 1901) and visualized with Principal Coordinates Analyses (PCoA). The effects of the same three factors (ecosystem management, extraction kit and sampling region) on fungal community composition were tested by using permutational multivariate analysis of variance (PERMANOVA) with 9999 permutations with the package vegan v2.5‐7.

We tested the effects of ecosystem management, extraction kit, and their interaction on binary or quantitative version of Jaccard compositional dissimilarities among samples, by constraining the permutations by sampling region. The natural logarithm of the total number of sequences per sample (sequencing depth) was introduced as the first fixed effect in all models.

The putative trophic mode of each ASVs was determined using the FUNGuild database according to Nguyen et al. (2016). Fungal functional guilds were assigned within the 12 most abundant guilds, namely animal endosymbiont, animal pathogen, endophyte, epiphyte, fungal parasite, lichen parasite, lichenized, plant pathogen, plant saprotroph, soil saprotroph, undefined saprotroph, and wood saprotroph. Only the guild assignments with “Probable” and “Highly probable” confidence rankings were accepted.

## 3 RESULTS

### 3.1 Leaf‐associated viral family richness and community composition differ in different environments

Based on normalized data, more viral reads and viral contigs were detected for the Cul‐2 site followed in turn by the Cul‐1, Nat‐2, and Nat‐1 sites (Table 1). According to the Blast‐based annotation, a total of 17 unique viral families were discovered from the four sites with respectively 15 and 14 viral families for Cul‐1 and Cul‐2, but only 7 and 11 viral families for Nat‐1 and Nat‐2 (Table 1). The family‐level richness of the virome thus appears to be higher for the cultivated than for the natural sites, an observation associated with the absence of several single‐stranded RNA (ssRNA) viral families (*Bromoviridae*, *Secoviridae*, *Virgaviridae* and *Benyviridae*) and of the *Caulimoviridae* pararetroviruses from the natural sites (Figure S2). On the contrary, the dsRNA virus families *Partitiviridae* and *Totiviridae* and ssRNA families *Endornaviridae*, *Alphaflexiviridae*, and *Tombusviridae* were present in all four sites. A high number of reads from *Endornaviridae* members were discovered in all viromes, especially those of the Cul‐2 and Nat‐2 sites (Bergerac; Figure S2).

**TABLE 1:** Main characteristics of the leaf‐associated viral and fungal communities in two cultivated and two natural ecosystems located in two regions (Bordeaux and Bergerac)

Site	Cul‐1	Cul‐2	Nat‐1	Nat‐2
Ecosystem management	Cultivated	Cultivated	Natural	Natural
Region	Bordeaux	Bergerac	Bordeaux	Bergerac
No. of plant species	40	33	34	29
*Leaf‐associated viral communities*				
Reads in viral contigs	289,019	651,841	106,501	234,088
% reads in viral contigs	22.8%	51.4%	8.4%	18.4%
*Blast annotation* ^a^				
No. of viral families	15	14	7	11
*RdRp*‐*OTU classification* ^b^				
Total no. of OTUs	73	50	26	55
Viral families^c^	13	12	5	9
Putative novel OTUs^d^	90.0%	83.7%	88.0%	96.3%
*Leaf‐associated fungal communities*				
Total unique ASVs	351	329	774	614
Average number of ASVs per multi‐species pool (mean ± SD)	114 ± 25	120 ± 18	221 ± 36	190 ± 41
Average Inverse Simpson per multi‐species pool (mean ± SD)	11.4 ± 4.3	6.0 ± 2.7	17.1 ± 5.0	19.9 ± 7.1

^a^ Blast annotation: the normalized sequences were assembled into contigs which were annotated using BlastN and BlastX against the non‐redundant nucleotide (nt) and protein (nr) GenBank databases with a conservative e‐value cut‐off of 10^−4^.

^b^ RdRp‐OTU classification: a search for RNA‐dependent RNA polymerase (RdRp) conserved motifs was performed on all assembled contigs and those encoding a viral RdRp were retrieved, aligned with reference sequences and clustered into different operational taxonomic units (OTUs) diverging more than 10%.

^c^ The lower number of families identified for each site using the OTU‐based approach results from the constraint that any virus for which the RdRp core‐encoding region is missing (due to incomplete genome coverage) will not be considered by this approach.

^d^ Putative novel OTUs: no RdRp‐encoding sequence in GenBank full‐filled the identity criterion (>=90% nt or aa identity) to be included in the corresponding OTU.

**FIGURE 2:**
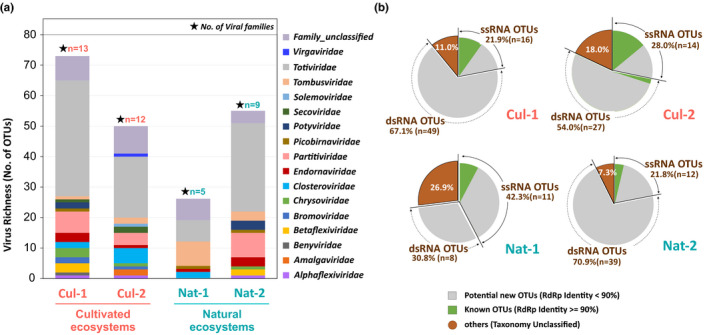
Leaf‐associated RdRp Operational Taxonomy Units (OTUs), virome composition, and known/novel status of RdRp OTUs at each sampling site. (a) Virome composition based on family level OTUs annotation. (b) Pie charts showing the proportion of ssRNA, dsRNA, and unclassified OTUs. Colors separate in each group the known viral OTUs (in green) for which a RdRp with >=90% identity was identified in GenBank and the potentially novel viral OTUs (in grey)

Based on viral RdRp clustering analyses, a total of 190 unique viral RdRp OTUs representing 16 viral families were identified from the study sites (Table S4). 73, 50, 26 and 55 OTUs representing 13, 12, 5, and 9 viral families were, respectively, discovered from the Cul‐1, Cul‐2, Nat‐1, and Nat‐2 sites, confirming the higher viral family richness in the cultivated sites (Table 1). For each site, many OTUs were identified as *Totiviridae* members except for the Nat‐1 site (Figure 2a). The virome of each site was generally structured with a large proportion (54.0% to 70.9%) of dsRNA viral OTUs and a smaller fraction of ssRNA viral OTUs (21.8% to 28.0%), except for the Nat‐1 virome in which the situation was reversed (42.3% ssRNA OTUs vs 30.8% dsRNA ones). Between 7.3% and 26.9% of OTUs could not be annotated by Blast at family level, depending on the site (Figure 2b). Overall, a large fraction of the detected OTUs (83.7% to 96.3%) putatively corresponds to novel viruses because no GenBank RdRp‐encoding sequence fulfilled the identity criterion (>=90% nt or aa identity) to be included in the corresponding OTU (Table 1; Figure 2b). The majority of the OTUs for which a GenBank counterpart could be identified corresponds to ssRNA viruses (Figure 2b).

### 3.2 Taxonomic and functional composition of the leaf‐associated fungal communities

As we sequenced both ITS1 and ITS2 amplicons, statistical analyses were performed in parallel on these two datasets, providing similar results (see R Markdown documents available at https://doi.org/10.15454/JQPBOW). Only the results obtained for the ITS1 region are presented here because ITS1 amplification and sequencing recovered a greater number of assembled reads, a greater number of reads assigned to the fungal kingdom, a higher ASVs richness, and a higher proportion of ASVs successfully assigned at the class level (*p*‐value of paired test <0.001, Table S5).

Taking into account the 32 ITS1 libraries extracted with the PowerSoil and DNeasy kits, a total of 1454 unique ASVs comprising 5 phyla, 24 classes, 170 families, and 344 genera were observed (Table 1). In each multi‐species pool, Ascomycota and Basidiomycota dominated fungal communities with a relative abundance (RA) of 49.9% ± 19.3% and 50.1% ± 19.3%, respectively. At the class level, Dothideomycetes and Tremellomycetes dominated with a relative abundance of 36.1% ± 17.9% and 42.8% ± 19.3%, respectively.

**FIGURE 3:**
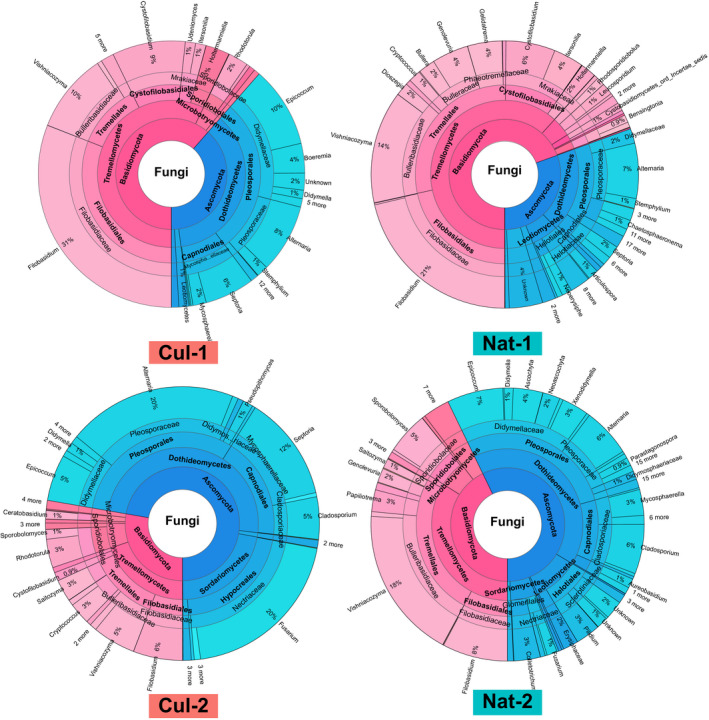
Krona chart of the taxonomic composition of leaf‐associated fungal communities in the four sampling sites. The taxonomic assignations are shown from Phylum down to Genus level

At each site, the Ascomycota and Basidiomycota also dominated the fungal communities (Figure 3). The Dothideomycetes and Tremellomycetes were generally the main classes followed by Sordariomycetes (22.1% at Cul‐2 site; Figure 3). Down to family level, Filobasidium had higher relative abundance in Cul‐1 and Nat‐1 sites followed by Alternaria and Fusarium in Cul‐2 site, and Vishniacozyma in Nat‐2 site (Figure 3).

Functional assignments using FUNGuild were successful for 923 out of 1517 ASVs recovered from both leaves and cultures, and 718 “highly probable” or “probable” guild assignments were accepted for the downstream analysis (Table S6). Taking into account solely the matched guilds in leaf samples, undefined saprotrophs (45.8% ± 13.7%) and plant pathogens (28.2% ± 8.4%) were the dominant guilds followed by animal pathogens (6.8% ± 3.1%) and endophytes (6.2% ± 2.1%) at each site (Figure S3). Plant saprotrophs and fungal parasites were more frequent in natural sites (Nat‐1 and Nat‐2), whereas animal endosymbionts were more frequent in cultivated sites (Cul‐1 and Cul‐2). The proportion of epiphytes was quite low (<1%) but relatively higher in natural sites than in cultivated ones (Figure S3).

### 3.3 Natural ecosystems are characterized by a greater fungal richness and diversity than cultivated ones

At site level, with a total of respectively 614 to 774 and 329 to 351 unique ASVs in natural (Nat‐2, Nat‐1) and cultivated (Cul‐2, Cul‐1) ecosystems, leaves from natural sites displayed twice as many fungal taxa as those from cultivated sites (Table 1). In addition, on average, more ASVs and higher multi‐species pools diversity values were discovered from natural sites than cultivated ones (Table 1).

**FIGURE 4:**
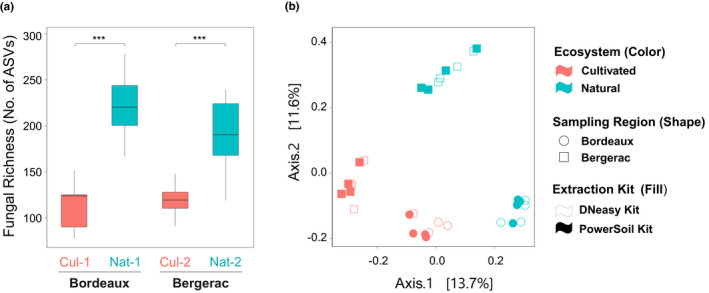
Fungal community richness and composition of leaves of wild plants from cultivated and natural sites. (a) Box plots illustrating fungal community richness associated to leaves collected in cultivated (red) and natural ecosystems (blue). Fungal richness was defined as the number of amplicon sequence variants (ASVs). Asterisks (***) identify a significant mean difference at an alpha level of .0001 for fungal richness by post‐hoc test. (b) Principal coordinates analysis (PCoA) of fungal communities associated to wild plant leaves. Dissimilarity among samples was calculated using the Jaccard binary index. Circles and squares respectively represent the Bordeaux and the Bergerac region; the filled and empty shapes respectively represent the fungal community obtained using PowerSoil and DNeasy DNA extraction kits; colors represent the Ecosystem management (red for cultivated and blue for natural)

Generalized Linear (Mixed) Models uncovered a significant effect of ecosystem management on fungal community richness and diversity (Table 2). Fungal richness and diversity of multi‐species pools were significantly greater in natural ecosystems as compared to cultivated ones (Figure 4a), and this increase was conditional on the geographic region sampled. The choice of DNA extraction kit in interaction with the ecosystem management also had a significant effect on fungal richness but not on fungal diversity.

**TABLE 2:** Generalized linear mixed‐effects models (GLMM) of fungal community richness and diversity among samples. Fungal richness was defined as the number of amplicon sequence variants (ASVs) per sample and fungal diversity as the Inverse of Simpson's diversity index. In all models, the effects of ecosystem management (Cultivated vs. Natural), DNA extraction kit (PowerSoil vs. DNeasy), and their interaction were tested as fixed effects and the sampling region (Bordeaux vs. Bergerac) as a random effect. Fungal richness and diversity were modeled with a Poisson distribution and log‐link function and a Gaussian distribution, respectively. Statistical significance (at 0.05 significance level) is highlighted in bold

Factors	GLMM of fungal community richness		GLMM of fungal community diversity	
	Chisq	*p*‐value	Chisq	*p*‐value
Ecosystem management (E)	**356.60**	**<0.0001**	**25.66**	**<0.0001**
Extraction kit (K)	0.55	0.46	0.03	0.86
E × K	**13.05**	**<0.001**	0.08	0.77

### 3.4 Leaf‐associated fungal community composition is shaped by ecosystem management and sampling region

PCoA analysis on Jaccard binary index suggested that the four sites had distinct community composition, and that the sites belonging to the same ecosystem management (natural versus cultivated) had more similar communities (Figure 4b). Permutational multivariate analyses of variance (PERMANOVA) of compositional dissimilarities indicated that the ecosystem management effect was significant (Table 3).

**TABLE 3:** Permutational multivariate analyses of variance (PERMANOVA) of compositional dissimilarities among fungal communities. Dissimilarities were estimated using the binary Jaccard distance. The total number of sequences per sample (sequencing depth, SD) was log‐transformed and introduced as the first explanatory variable in all models. The effects of ecosystem management (Cultivated vs. Natural), DNA extraction kit (PowerSoil vs. DNeasy) and their interaction were first tested by constraining the permutations by sampling region. Statistical significance (at 0.05 significance level) is highlighted in bold

Factors	*F*‐value	*R* ^2^	*p*‐value
Log (SD)	1.05	0.032	0.22
Ecosystem management (E)	**4.00**	**0.120**	**0.0001**
Extraction kit (K)	0.80	0.024	0.68
E × K	0.56	0.017	0.99

Venn diagrams of fungal ASVs of multi‐species pools within each sampling site showed a significant proportion of 32.2% to 42.6% (on average 36.8% ± 5.1%) of ASVs shared between at least two pools and a core of on average 12.9% ± 4.0% of ASVs shared between all pools of a sampling site (extremes 10.4–18.8%; Figure S4). When comparing the overall unique and shared ASVs among sites, a large proportion (74.2%) of fungal ASVs were site‐specific and only 5.4% were shared among all sites (Figure S5).

### 3.5 Fungal and viral communities of cultured fungal colonies following a culturomics approach

The culturomic approach recovered between 480 (Cul‐2) and 1279 (Cul‐1) fungal colonies (Figure S6). A total of 182 unique ASVs comprising 3 phyla, 9 classes, 48 families, and 77 genera were observed from the colonies using the ITS1 dataset, and 133 unique ASVs using the ITS2 dataset. Considering either ITS1 or ITS2 dataset, fungal communities from each site were generally dominated by Ascomycota with a relative abundance (RA) of 98.2% ± 1.6%, followed by tiny fraction of Basidiomycota. At the class level, Dothideomycetes dominated the culture‐associated fungal communities in cultivated sites (RA: 78.2% ± 5.7%) and accounted for a lower proportion of 23.4% ± 8.7% in natural ones. On the contrary, Leotiomycetes dominated the culture‐associated fungal communities in natural sites (RA: 69.9% ± 9.2%). At genus level, *Pilidium* consistently dominated in natural sites (RA: 63.3% ± 10.2%), whereas *Alternaria*, *Cladosporium*, and *Epicoccum* were more abundant in cultivated sites (RA ranging from 10.8% to 29.5%).

Despite the relatively large number of colonies cultivated in these experiments, only a small fraction of fungal ASVs identified from the leaf samples were also observed from fungal colonies (5.2% to 14.8% with an average of 9.5% ± 4.2% of ASVs inferred from ITS1 dataset and 9.9% to 20.9% with an average of 14.2% ± 5.1% for the ITS2 dataset) (Figure S6). Interestingly, a significant fraction of ASVs from fungal colonies were culture‐specific and were not detected by the metabarcoding performed on the plant samples (15.4% to 30.7% for the ITS1 dataset; 30.9% to 63.1% for the ITS2 dataset; Figure S6).

To evaluate the proportion of phytoviruses to mycoviruses in leaf samples, the non‐normalized datasets were used in order to maximize the probability of detecting shared viruses between the plant‐ and culture‐associated viral communities. Remarkably, the viromes obtained from the cultivated fungal pools were almost completely different from the viromes obtained from the plant pools. Based on the Blast annotation, the culture‐associated viromes collectively comprised 13 viral families (seven ssRNA families and six dsRNA families, Table S7). *Totiviridae*, together with *Chrysoviridae*, *Endornaviridae*, *Alphaflexiviridae*, and *Partitiviridae* members were detected from both leaf‐ and culture‐associated viromes. On the other hand, a range of viral families were only detected from the cultured fungi, including the *Gammaflexiviridae*, *Hypoviridae*, *Tymoviridae*, *Narnaviridae*, *Fusarividae*, and *Birnaviridae*. Leaf‐associated viromes were characterized by a greater number of viral families compared with culture‐associated viromes (Table S7). Compared with leaf‐associated fungal communities versus culture‐associated fungal communities, the viromes of leaves and fungal cultures were found almost totally different, with only two OTUs shared for the Cul‐2 site (out of a total of 29), whereas no shared OTU could be detected in the other three sites out of a total of 54 viral OTUs detected from the corresponding fungal cultures (Figure S6). The reciprocal mapping of the reads of one virome type against the contigs of the other type confirmed that only a very minor fraction of viruses was shared between the plant and fungal cultures‐derived viromes (data not shown).

## 4 DISCUSSION

In this study, we performed a large‐scale, joint analysis of the viral and fungal communities associated with wild plant populations to test two main hypotheses. The first tested hypothesis is that leaf‐associated viral and fungal communities are richer in natural ecosystems than in cultivated ones. Our results showed that leaf‐associated fungal communities of weeds and wild plants were indeed consistently richer in natural than in cultivated ecosystems. However, in contrast to our hypothesis, leaf‐associated viral communities were richer at family level in cultivated ecosystems, suggesting that different processes and forces shape the viral and fungal communities associated to plant leaves. Differences in dispersion mechanisms between fungi and viruses or the contrasted impact of fungicide treatments on fungal and viral communities are certainly among potential drivers. Leaf‐associated fungal communities are partially acquired through horizontal transmission (Lemanceau et al., 2017). Horizontally transmitted fungi are transported from one plant to the other by wind, insects, irrigation water, and rain. Therefore, the lower diversity of the fungal communities of cultivated crops (Compant et al., 2019) may have affected that of the weeds and wild plants growing nearby. Leaf‐associated fungal communities are also impacted by fungicides (Bertelsen et al., 2001; Moulas et al., 2013) and fungicide treatments applied in cultivated sites may also have reduced fungal diversity on the weeds and wild plants growing near the crops. These two hypotheses are not mutually exclusive. Our results on viral richness parallel those of Bernardo et al. (2018) who also observed a higher family‐level virus diversity in cultivated areas. This difference was however less clear‐cut in the present study when defining viral richness as the number of OTUs, which represents a proxy for viral species. Domestication and cultivation, by reducing plant species diversity, have been suggested to be responsible for increased viral infections in cultivated ecosystems (Roossinck & García‐Arenal, 2015). Such an effect may also have contributed to the results reported here if spill‐over of frequent viruses in crops contributes a significant share of the virus communities of weeds/wild plants growing side‐by‐side with the crops.

Our results also confirmed the second tested hypothesis that ecosystem management influences the composition of fungal and viral communities in leaf samples. Ecosystem management accounted for about 12% of the explained variance in fungal community composition, and was associated with the presence‐absence of viral families. For instance, several single‐stranded RNA (ssRNA) virus families and the *Caulimoviridae* pararetroviruses were absent from the natural sites. In addition, our analyses also showed a strong site‐specificity of leaf‐associated fungal and viral communities (Figure S5). The majority (74.2%) of fungal ASVs was detected solely in one site, whereas the fraction of viral OTUs unique to each site was significantly higher (93.2% of viral OTUs were site‐specific). These site‐specific fungal ASVs explain the clustering in the PCoA analysis that groups together different plant pools from a given site and unambiguously separate them from other sampling sites. These variations are potentially associated with host plant identity because 55% sampled plant species were specific to one of the sampling sites. Besides, local environmental conditions may contribute to the observed site‐specificity. As reviewed in Vacher et al., (2016), environmental conditions such as relative humidity, wind speed, and temperature have been recognized to significantly affect the assemblage of foliar fungi. The higher site‐specificity of leaf‐associated viral communities is also potentially affected by sampled plant species identity. It should however be considered that this high site specificity of viral populations is potentially only valid for viruses present at a high frequency in the sampled plant populations. Indeed, with only 5–7 individual plants sampled per species (200 plants per sampling site), the ability to detect viruses with a low, less than 10% prevalence in the sampled species, is limited. It is therefore possible that deeper sampling of each plant species, involving more numerous individuals may in the future provide a different picture by making it possible to take low‐prevalence viruses into account.

Besides testing the two above hypotheses, we tried in this study to evaluate the ratio of phytoviruses versus mycoviruses in leaf samples. The results remain inconclusive because the viral communities associated with plant samples and fungal cultures, although derived from the same initial plant samples, were remarkably different. This incongruence between plant‐associated and culture‐associated viral communities at the OTU level is in contrast with some observations, in particular those reported by Al Rwahnih et al. (2011) in which a limited culturomics effort, involving only 11 fungal colonies, allowed to demonstrate a mycovirus status for 5 of the 25 (20%) viruses identified in a grapevine virome. One possible hypothesis to explain these differences may be linked to the pooling strategy used here, which, while allowing the analysis of many more individual samples, may favor the detection of highly prevalent or high concentration viruses. On the other hand, fungal culturomics of plant leaves has made clear that in vitro culture‐based approaches grossly underestimate fungal diversity (Roossinck, 2015b), and the results reported here are in line with this general observation with less than 15% of the fungal ASVs associated to leaves recovered in fungal cultures. However, it is noteworthy that the culturomics provided a significant fraction of cultivated fungi ASVs (14.3% to 37.5%) or of viral OTUs that were not detected during the direct analysis of plant samples, thus highlighting the incompleteness of both methods. In this respect, further efforts are clearly needed to better understand the links between the mycovirus communities and phytovirus communities.

## 5 CONCLUSIONS

The results presented here provide a large‐scale parallel analysis of the viral and fungal communities associated with complex plant populations in cultivated and natural ecosystems. Ecosystem management had a significant influence on the microbial communities in our study, characterized by a higher fungal community richness in natural ecosystems and a higher viral family richness in cultivated ones. These results suggest that leaf‐associated fungal and viral communities are under the influence of different ecological drivers. In addition, the leaf‐associated fungal and viral community compositions of the four sites showed a strong site‐specificity. These observations clearly deserve further confirmatory efforts, for example by increasing sample size (individual plants/species) and sampling sites with categorized environment conditions.

## CONFLICT OF INTEREST

The authors declare no conflict of interest.

[Correction added on 18 June 2021, after first online publication: Conflict of Interest statement added to provide full transparency.]

## AUTHOR CONTRIBUTION

Thierry Candresse and Armelle Marais designed the experiments and supervised the progress, Corinne Vacher provided many useful suggestions for ITS sequencing. Yuxin Ma performed the molecular experiments, data analysis, and interpretation. Tania Fort provided R scripts and contributed to the data processing of fungal ITS datasets. YM and TC wrote the manuscript; TF, CV, and AM with other authors provided critical reading of this manuscript and its further improvement. Marie Lefebvre and Sébastien Theil developed the VirAnnot pipeline and performed the viral sequence processing and OTU annotation.

## Supporting information

Fig S1‐6

Table S1‐7

## Data Availability

The cleaned sequence reads in each library for viral community analysis and the demultiplexed sequences obtained from the sequencing of ITS1 and ITS2 amplicons have been deposited on the INRAE National Data Portal under the identifier https://doi.org/10.15454/X23KJF. The fungal ASV table, sample metadata table, taxonomy table, and scripts of bioinformatic and statistical analyses are available at https://doi.org/10.15454/JQPBOW.
