# Supplementary material for: Leaf‐associated fungal and viral communities of wild plant populations differ between cultivated and natural ecosystems
Source: Plant Environ Interact. 2021 Mar 25;2(2):87–99. doi: 10.1002/pei3.10043 (PMC10168098; doi:10.1002/pei3.10043)
Supplement: Supplementary file 1 — Fig S1‐6 [file PEI3-2-87-s001.docx]

**
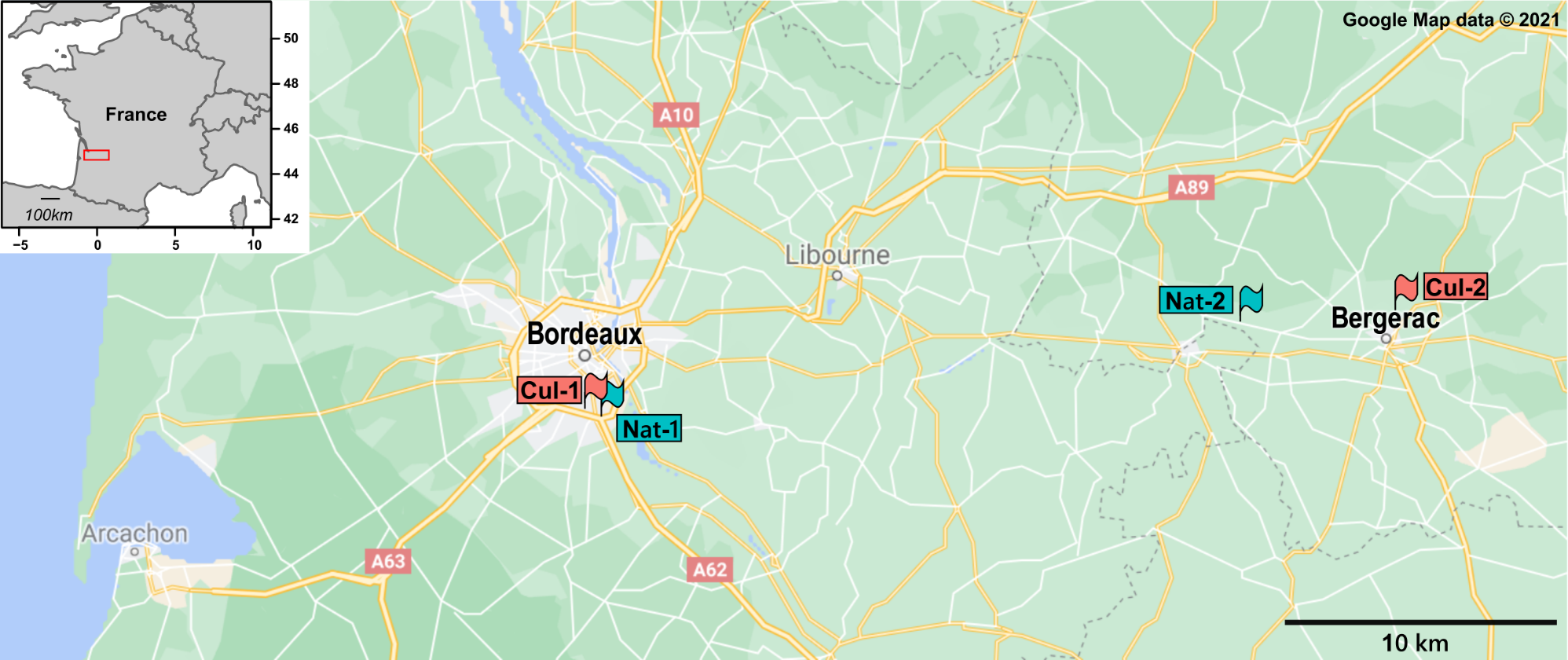
**

**Supporting Information Figure S1** **Geographic position of the four sampling sites represented by flags in red for cultivated sites (Cul-1 and Cul-2) and in blue for natural sites (Nat-1 and Nat-2).** Cul-1 and Cul-2 were cultivated, horticultural agro-ecosystems in which vegetable crops were grown. The Cul-1 site harbored a large range of crops, including lettuce, spinach, pepper, turnip, while the Cul-2 site mostly had carrots. Nat-1 and Nat-2 were natural, dry grasslands.


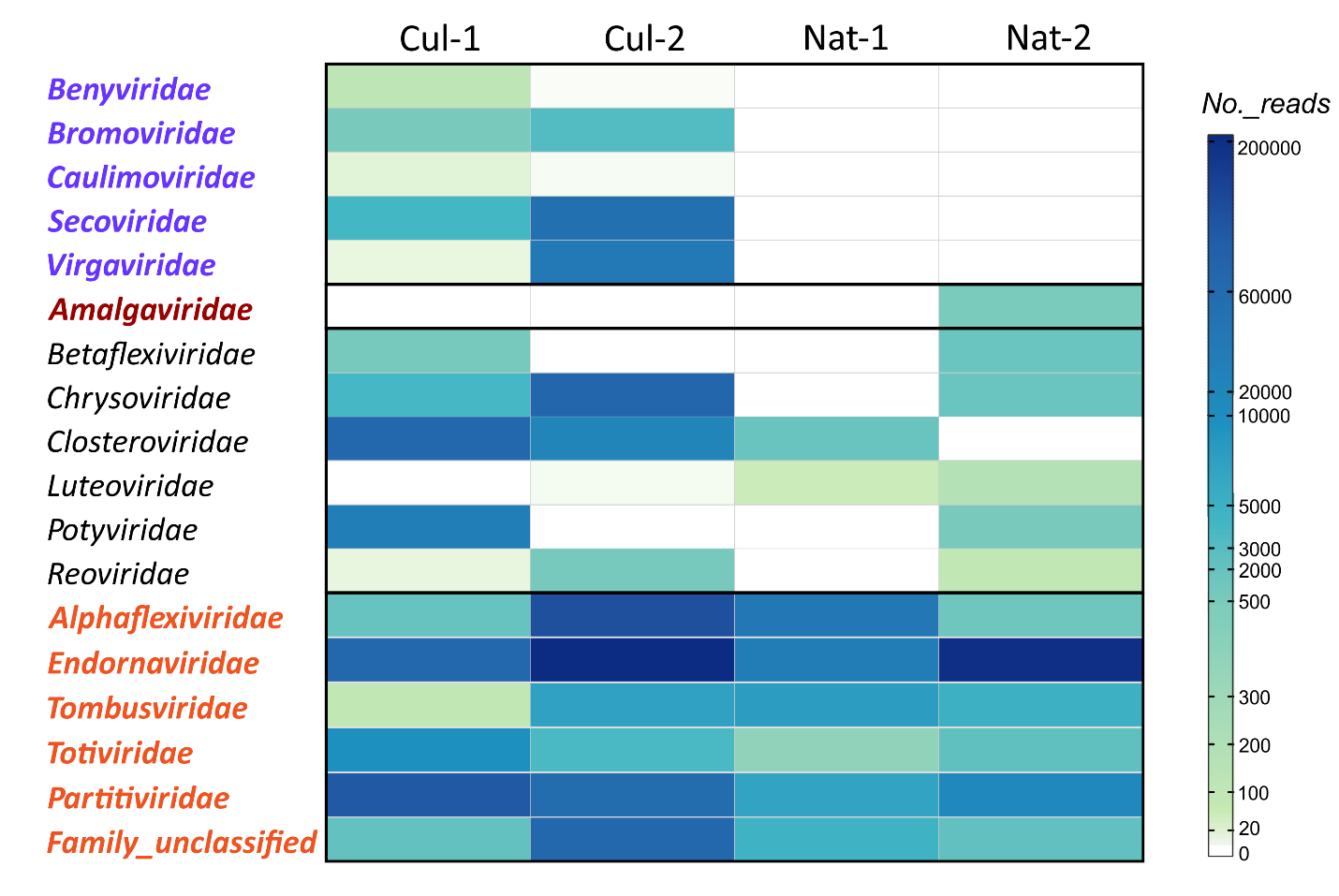


**Supporting Information Figure S2** Heatmap showing the number of reads corresponding to 17 viral families identified from leaf samples in each sampling site, as estimated from the results of BlastN and BlastX analyses. The cultivated (Cul-1 and Cul-2) and natural ecosystems (Nat-1 and Nat-2) are indicated on the upper side and viral families are on the left side. Viral families are color-coded blue (specific in cultivated sites), ruby (specific in natural site), black (in both cultivated and natural ecosystems) and orange (in all sampling sites). Cell color intensity is proportional to the number of reads, following the scale on the right.

**
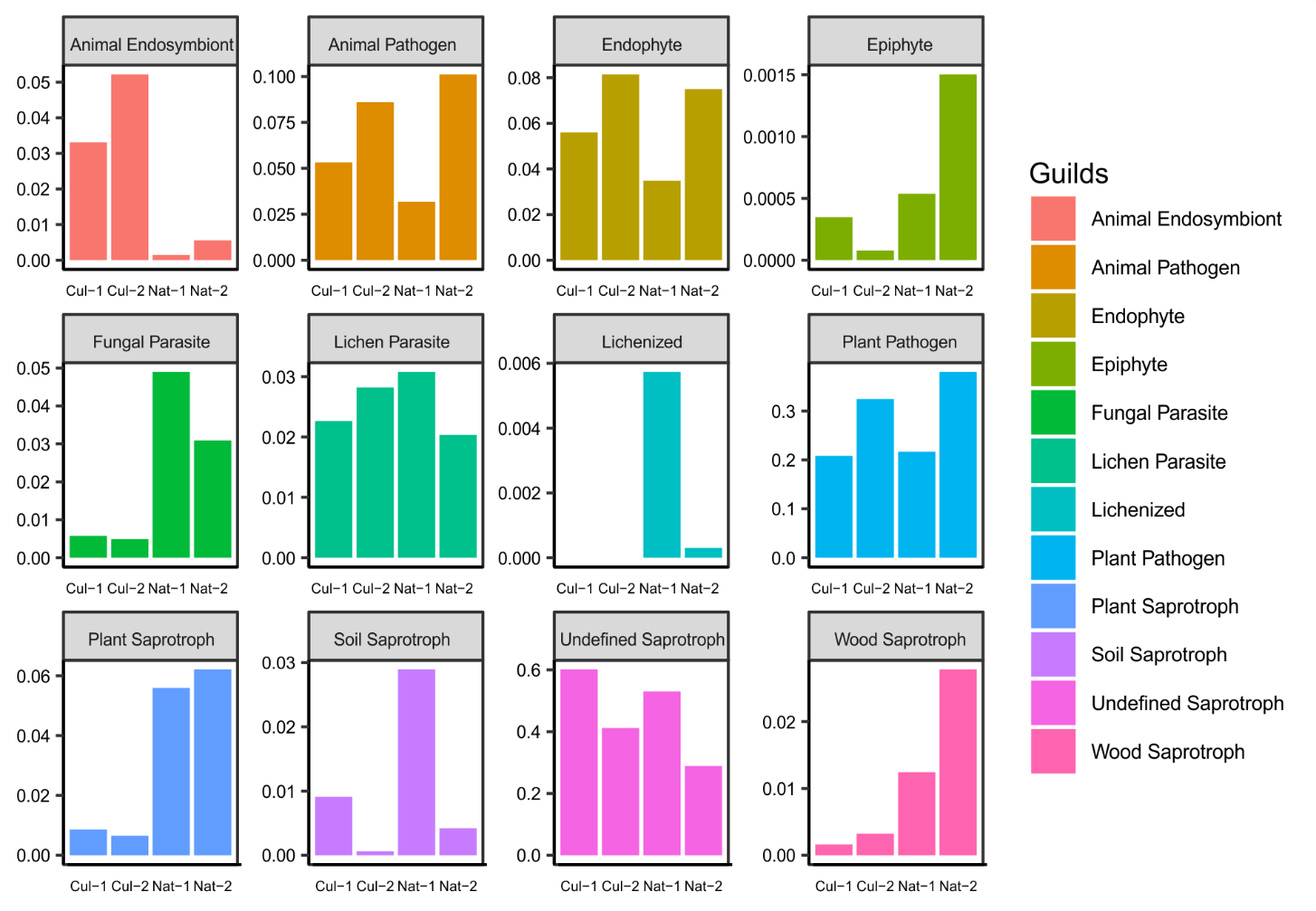
**

**Supporting Information Figure S3 Functional profile of fungal communities associated to leaf samples.** Each color and barplot represent a putative function of leaf-associated fungal ASVs inferred using FUNGuild. The barplots indicate the relative abundance (%) of a putative function for each site (cultivated sites: Cul-1 and Cul-2; natural sites: Nat-1 and Nat-2).

**
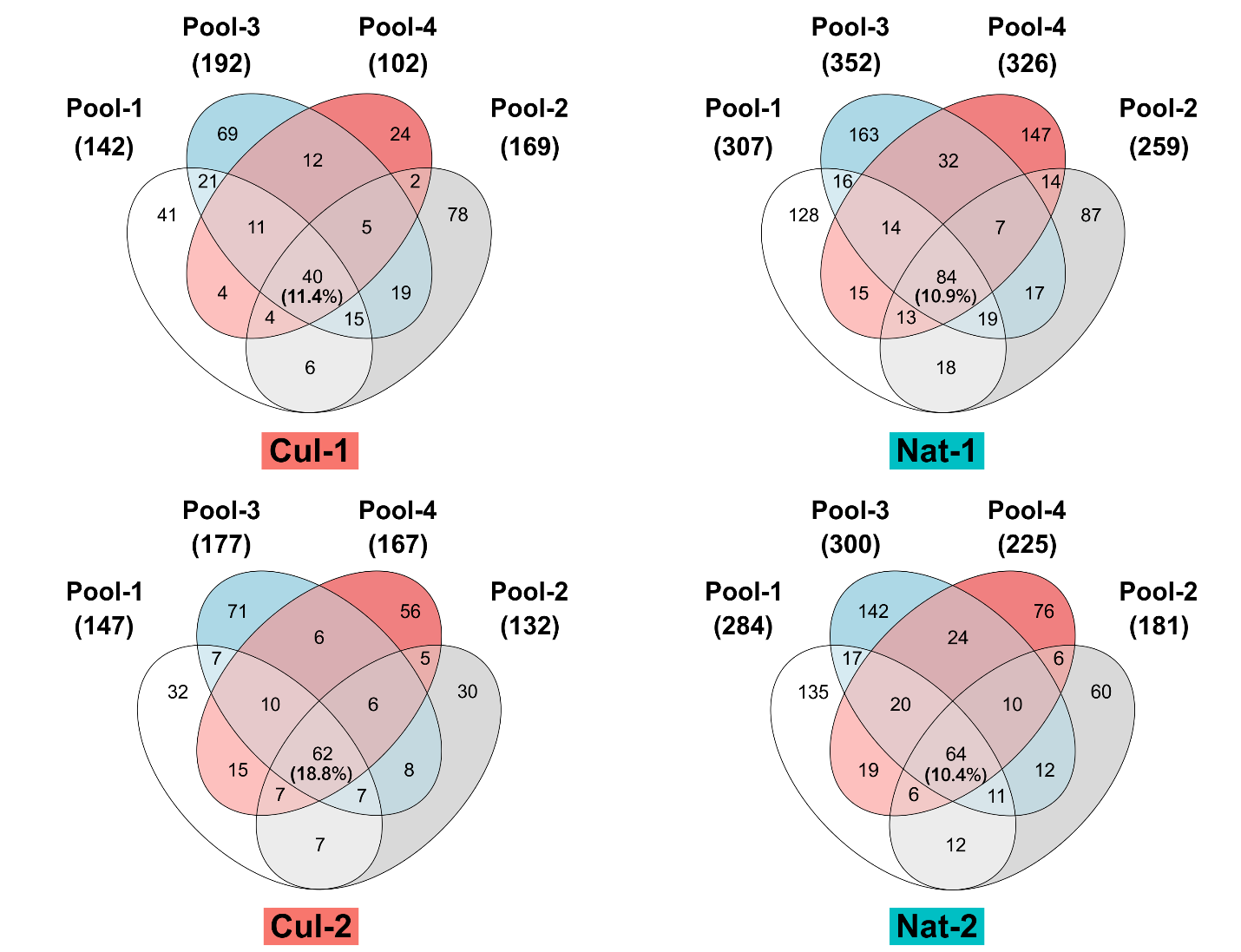
**

**Supporting Information Figure S4** Venn diagrams showing the shared fungal ASVs between different plant pools from the same sampling site. ASVs of cultivated (Cul-1 and Cul-2) and natural ecosystems (Nat-1 and Nat-2) were determined for four pools of wild plant leaves (pool-1, pool-2, pool-3 and pool-4). The total number of unique ASVs of each pool in parenthesis, shared ASVs among the 4 pools (black numbers) and the percentage of core ASVs in each sampling site are indicated.


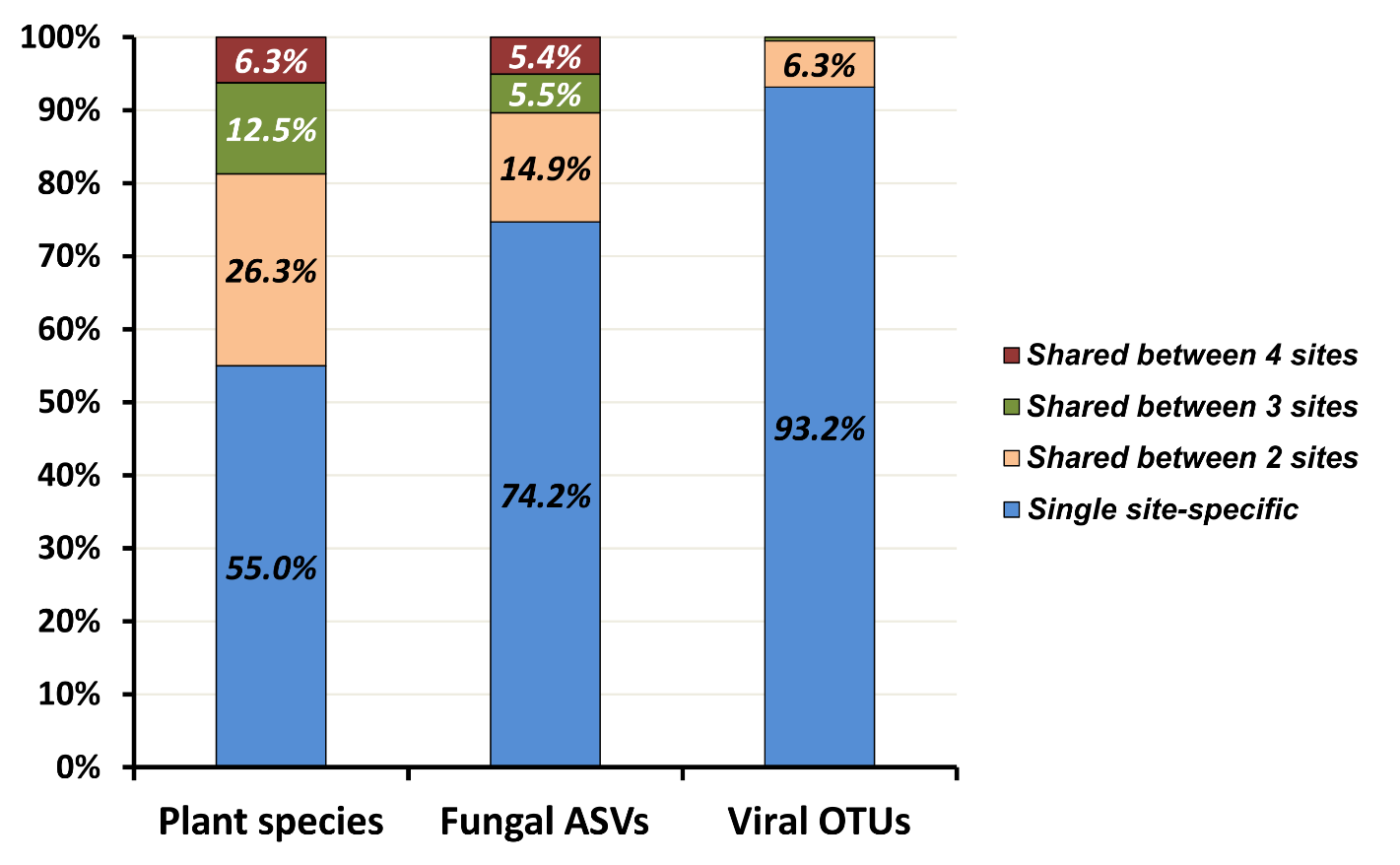


**Supporting Information Figure S5** Bar plot showing the frequency of sampled plant species, detected fungal ASVs and detected viral OTUs between the four study sites.

**
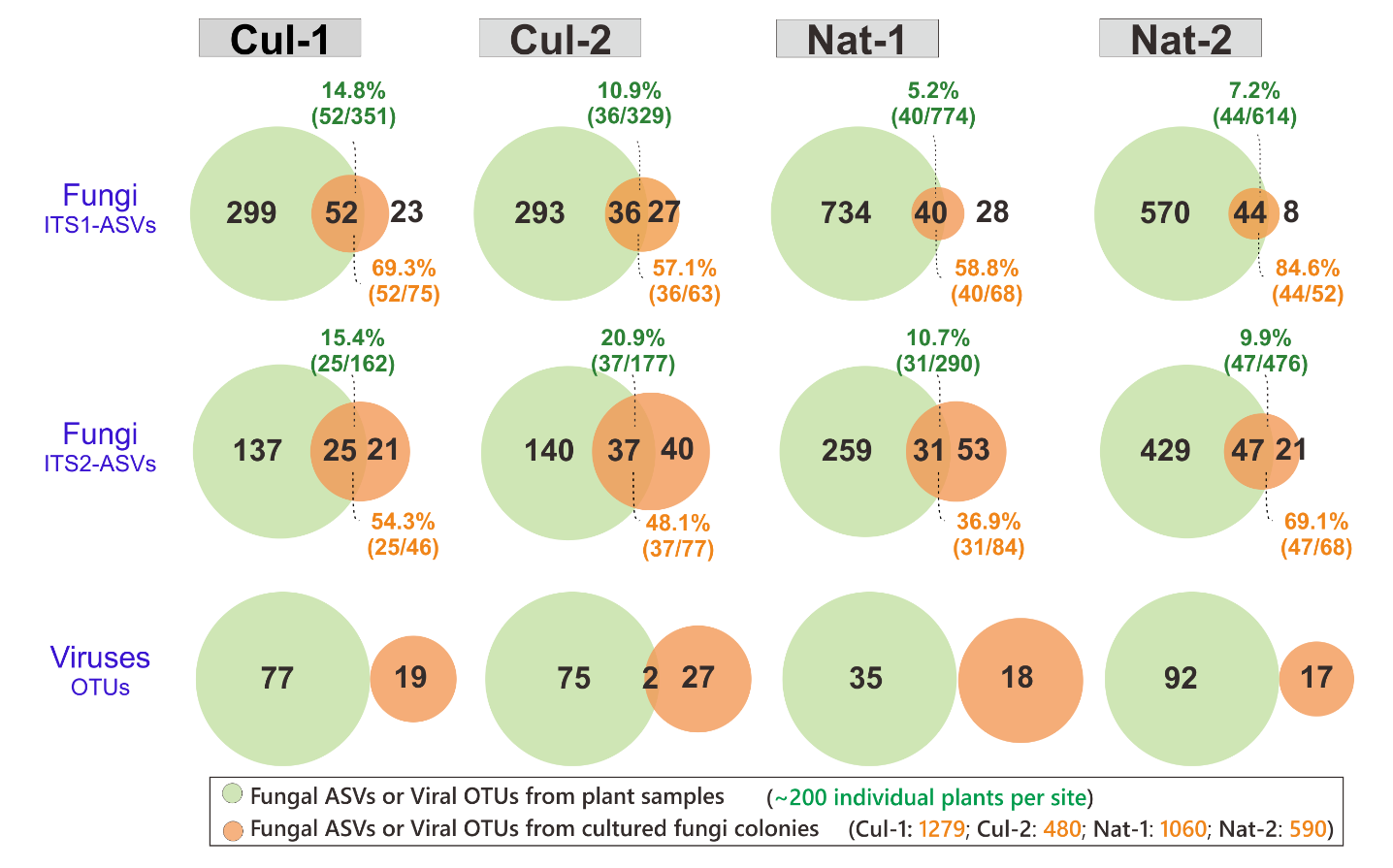
**

**Supporting Information Figure S6** Scaled venn diagrams showing the number and proportions of shared fungal ASVs and viral OTUs between leaves and fungal colonies for each sampling site. The green values in the upper part represent the proportion of leaf-associated fungal ASVs that are also found in fungal colonies. Orange values in the lower portion represent the proportion of fungal ASVs from fungal colonies that are also associated to leaves.
